# Cost of illness analysis in individuals with overweight or obesity and chronic low back pain in the Bern metropolitan area (the BO2WL trial)

**DOI:** 10.3389/fpubh.2025.1705891

**Published:** 2026-01-13

**Authors:** Alexander P. Schurz, Nathanael Lutz, Melanie Liechti, Anneleen Malfliet, Matteo Vanroose, Zoë Maebe, Jo Nijs, Wouter Van Bogaert, Peter Clarys, Ron Clijsen, Heiner Baur, Jan Taeymans, Tom Deliens

**Affiliations:** 1 Department of Movement and Sport Sciences, Faculty of Physical Education and Physiotherapy, Vrije Universiteit Brussel, Brussels, Belgium; 2 Department of Health Professions, Bern University of Applied Sciences, Bern, Switzerland; 3 Faculty of Medicine, University of Bern, Bern, Switzerland; 4 Pain in Motion Research Group (PAIN), Department of Physiotherapy, Human Physiology and Anatomy, Faculty of Physical Education and Physiotherapy, Vrije Universiteit Brussel, Brussels, Belgium; 5 Pain in Motion International Research Group, Brussels, Belgium; 6 Department of Physical Medicine and Physiotherapy, Chronic Pain Rehabilitation, University Hospital Brussels, Belgium; 7 Research Foundation—Flanders (FWO), Brussels, Belgium; 8 PijnPraxis.be Private Practice for Pain Physiotherapy, Leopoldsburg, Belgium; 9 Department of Health and Rehabilitation, Unit of Physiotherapy, Institute of Neuroscience and Physiology, Sahlgrenska Academy, University of Gothenburg, Gothenburg, Sweden; 10 Department of Business Economics, Rehabilitation and Exercise Science Laboratory RESLab Health, and Social Care, University of Applied Sciences and Arts of Southern Switzerland, Manno, Switzerland; 11 International University of Applied Sciences THIM, Landquart, Switzerland; 12 Faculty of Human Sciences, Institute of Sport Science, University of Bern, Bern, Switzerland; 13 Department of Physiotherapy, Human Physiology and Anatomy, Faculty of Physical Education and Physiotherapy, Vrije Universiteit Brussel, Brussels, Belgium

**Keywords:** cost of illness, global burden of disease, low back pain, multimorbidity, obesity, overweight, Switzerland

## Abstract

**Background:**

Chronic low back pain (CLBP) imposes substantial societal costs, with comorbid overweight or obesity possibly further increasing them. This study assessed the cost of illness of individuals with CLBP and comorbid overweight or obesity in the Bern metropolitan area (Switzerland) from a societal perspective.

**Methods:**

Following the Consolidated Health Economic Evaluation Reporting Standards 2022 and using preliminary baseline data from a randomised controlled trial, societal costs, including direct and indirect costs, over 3 months were calculated for adults with overweight or obesity and CLBP. Self-reported data were monetised using a bottom-up, prevalence-based approach, and absenteeism and presenteeism were valued via the human capital approach. Mean costs with bootstrapped 95% confidence intervals (CI) were reported in 2025 Swiss Francs (CHF) per cost category.

**Results:**

Fifty-three individuals (19 male, 34 female) with a mean age of 47.8 years (SD: 10.1) were included, comprising 18 individuals with overweight and 35 with obesity. Mean direct medical costs were CHF 543.69 (95% CI = [382.80; 686.33]), and mean direct non-medical costs were CHF 19.39 (95% CI = [0.00; 38.63]) respectively. Indirect costs accounted for 80% of total societal costs, with costs attributable to absenteeism of CHF 1,000.39 (95% CI = [2.63; 1,681.40]), presenteeism costs of CHF 549.47 (95% CI = [281.67; 774.67]) and costs due to impaired unpaid work of CHF 745.56 (95% CI = [241.02; 1,436.80]). Total societal costs averaged CHF 2,850.44 (95% CI = [1,667.17; 3,829.17]).

**Conclusion:**

Despite the exclusion of individuals with uncontrolled, untreated comorbidities and those receiving invalidity payments, this study demonstrates a substantial financial burden of individuals with overweight or obesity and CLBP. From societal perspective, this burden is primarily driven by productivity losses. These findings emphasise the need for targeted cost-effective clinical interventions, coordinated policy initiatives, and further research to mitigate the significant societal impact of this population.

**Trial registration number:**

NCT05811624 (Clinicaltrials.gov). Acronym: BO2WL trial.

## Introduction

In Switzerland (CH), healthcare expenditure has doubled since 2000, reaching 91.5 billion Swiss Francs (CHF) in 2022 (€61.5 billion), with further increases expected in the upcoming years (1). This corresponds to mean monthly healthcare costs per person of around CHF 526 (€354) (1). Among the Organisation for Economic Co-operation and Development (OECD) countries, CH ranked fourth in healthcare expenditure as a proportion of gross domestic product (GDP) at 11.7%, following the United States (16.6%), Germany (12.7%) and France (12.1%) (2). Most of the healthcare costs were attributed to inpatient (CHF 19.6 billion, € 13.2 billion) and outpatient curative care (CHF 19.2 billion, €12.9 billion), long-term care, and support services (CHF 18.4 billion, €12.4 billion) (1). Expenditures on healthcare goods, such as medications or therapeutic treatments, accounted for CHF 14.6 billion (€ 9.8 billion) (1).

These costs highlight the importance of understanding the economic burden of specific diseases to prioritise the allocation of healthcare resources effectively. Based on an evaluation of disease specific-healthcare spending in CH in 2012 and 2017, the most expensive disease groups were mental, musculoskeletal (MSK) and neurological disorders (3). Among the MSK disorders the increase in healthcare expenditure between 2012 and 2017 was mostly related to outpatient care (60.7%) and inpatient long-term care (24.1%) (3). Of the MSK disorders, osteoporosis, osteoarthritis, rheumatoid arthritis and low back pain (LBP) were the main cost contributors (3).

In CH, 1.2 million individuals were affected by LBP in 2021 (4), leading to CHF 3.9 billion (€2.6 billion) in direct medical costs with productivity losses accounting for CHF 7.2 to 9.9 billion (€4.8 to €6.6 billion), relating to 78% of the total societal costs (5). Half of the people with LBP used minimal healthcare resources, while the others spent vast amount of money on pain relief (5). This aligns with findings from a systematic review summarising cost-related data from other European studies (*n* = 12), the United States (*n* = 5), and the Western Pacific region (*n* = 4) (6). Annual direct medical costs ranged from CHF 3.4 to 3.9 billion (€2.3 to €2.6 billion) on a population level (6). Patient-level cost data from a healthcare payer perspective for 6 months ranged from CHF 1,239 (95% CI = [1,115.5; 1,381.5]; €715.6, 95% CI = [644.2; 797.8]) in France (7) to CHF 1,827.8 (95% CI = [1,545.1; 2,203.7]); €1,002.0 (95% CI = [847.0; 1,208.0]) in Germany (8). An important economic burden arose from absenteeism (absence from work due to LBP) and presenteeism (reduced productivity during work due to the health situation) (9). For example, in Sweden, one LBP episode amounted to CHF 4,344 (€2,753) per capita from a societal perspective, with 66% due to work absenteeism (10), while in Serbia, 90% of societal costs were linked to productivity loss (both absenteeism and presenteeism) in individuals with LBP (11).

Chronic low back pain (CLBP), characterised as persistent LBP for more than 12 weeks (12), exacerbates the economic burden of LBP, with higher pain intensity linked to more absenteeism and presenteeism (13). In Germany, total societal costs for 6 months were calculated at CHF 3,265.0 per patient (95% CI = [2,681.6; 4,017.0]; €1,789.8, 95% CI = [1,470.0; 2,202.0]) in 2004 which was almost twice the amount of acute LBP (8). The risk of CLBP increases with higher body mass index (BMI) in both sexes especially in individuals with BMI values from 30.0 to 34.9 kg/m^2^ with risk ratios of 1.39 (95% CI = [1.23; 1.58]) for women and 1.37 (95% CI = [1.15; 1.64]) for men when compared to normal weight (14).

In CH, recent population-based studies have evaluated the cost of illness of individuals with overweight or obesity, including seven comorbidities such as asthma or depression (15, 16). However, CLBP was not considered in these evaluations. This omission is noteworthy, since previous research revealed that overweight or obesity are related to higher rates of health care seeking for LBP (17).

Therefore, the present study addressed this knowledge gap by evaluating the total costs from a societal and healthcare payer perspective for individuals with CLBP who are overweight or obese in the metropolitan area of Bern (CH).

## Materials and methods

This manuscript adheres the Consolidated Health Economic Evaluation Reporting Standards 2022 (CHEERS 2022) guideline (18) and the paper by Jo (19) considering relevant criteria for cost reporting. The Swiss baseline data of The Back Pain Overweight Obese Weight Loss Study (BO2WL) served as the basis of this cost of illness study, which is a randomised controlled trial with an embedded health economic evaluation conducted in CH (Clinicaltrials.gov: NCT05811624). The cost of illness was evaluated using a prevalence-based, bottom-up approach. The study protocol and health economic analysis plan of the BO2WL trial have been published elsewhere (20, 21). The CH part of the BO2WL trial, and therefore this cost of illness study received local ethical approval in the Canton of Bern, CH (BASEC number: 2022–02210).

### Study participants

Study participants were recruited through the BO2WL trial and data from baseline measurements conducted from May 2023 to November 2025 in CH were included in this cost of illness study. The recruitment strategies for this cost of illness study and the BO2WL trial involved medical institutions (e.g., general practitioners and physiotherapists), local newspapers, and social media (20, 21). Table 1 shows an overview of the main inclusion and exclusion criteria for the individuals with overweight or obesity and CLBP.

**Table 1:** Eligibility criteria for the BO2WL trial.

Criteria	Inclusion criteria	Exclusion criteria
Language	Good knowledge of German language (verbal and written)	
Age	18 to 65 years	
BMI	Overweight: ≥25 to <30 kg/m^2^	Healthy body fat percentage
	Obese: ≥30 to <40 kg/m^2^	Healthy weight-to-hip-ratio (sex and age adjusted)
Chronic low back pain	Chronic non-specific low back pain according to National Institutes of Health (65): Pain duration: >3 months ≥50% of the days in past 6 months	Specific spinal pathology (e.g., disc herniation with clear neuropathic component, spinal stenosis, spondylolisthesis)
Leg pain	< 7/10 on the NRS	
Other diagnoses		Severe underlying diseases (e.g., metabolic disease, diabetes, cancer, ongoing severe depression, cognitive impairment, eating disorder) Pregnancy or post-partum in the preceding year, breast-feeding
Current treatment	Continuation of usual care 6 weeks prior and during study participation (i.e., steady state in usual care)	Dietary or exercise intervention in the last 6 weeks or currently ongoing
Other criteria	Study participation in Bern (CH) Signed informed consent	In clarification of invalidity payments

BMI, body mass index; CH, Switzerland; NRS, numeric rating scale.

Individuals were recruited in the Bern metropolitan area (CH) and assessed at Bern University of Applied Sciences (Bern, CH). The BO2WL study aims to evaluate the (cost-)effectiveness of adding a lifestyle intervention to pain neuroscience education (PNE) and Cognition-Targeted Exercise Therapy (CTET) in the treatment of individuals with overweight or obesity and CLBP, compared to PNE and CTET alone (20, 21). Participants were randomised to either the intervention group (i.e., PNE, CTET, plus an additional lifestyle intervention) or the control group (i.e., PNE, CTET) (20, 21). The lifestyle intervention, which was only implemented for the intervention group, consisted of physical activity promotion and a dietary intervention to support weight reduction (21). The intervention lasted 14 weeks with measurements being conducted 1 week prior and after the intervention period as well as 3-, 6-, 9-, and 12-months post therapy completion. Any measurement timepoint aside from baseline were not considered for the present paper. At time of baseline measurement, randomisation status was unknown to both the assessor and the study participant, as both were blinded. All participants signed the informed consent form prior to participating in the study.

### Data collection

Cost-related data were collected retrospectively for the last 3 months during the BO2WL trial baseline measurements via validated questionnaires. Units consumed for direct medical (e.g., visits to physicians, specialists, physiotherapists, hospital stays, medications) and direct non-medical costs (e.g., formal or informal care, transportation) were gathered via the translated German version of the Medical Consumption Questionnaire (iMCQ) (22), while units consumed regarding indirect costs (e.g., short-term absenteeism, presenteeism, unpaid work) were collected via the Productivity Cost Questionnaire (iPCQ) (23, 24). Indirect medical costs as well as intangible costs, were not considered due to the short time horizon of 3 months and to avoid double-counting (25–27).

In addition to these cost-related data, self-reported baseline demographic data were also collected, and include sex, age, BMI, marital status, employment status, educational level, average weekly physical activity (in minutes), and duration of CLBP (in months). Furthermore, quality of life was assessed using the European Quality-of-Life-5 Dimensions-5 Levels (EQ-5D-5L) instrument (28). Health states were gathered from the questions on the five dimensions: mobility, self-care, usual activity, pain/discomfort and anxiety/depression. Health states were transformed into health utility scores using a mapping function (29) and the German tariff value set (30). At present, CH does not have a country-specific value set to calculate health utilities. Of the tariff value sets currently available, the German value set is the closest aligned with CH’s sociodemographic context. Health utilities range from 1, corresponding to perfect health, to 0, which represents the state of death (29). The EQ-5D-5L also included the self-reported subjective health status, which was assessed using a numerical analogue scale ranging from 0 to 100, with higher scores indicating a better health status. All collected data were filled out online using the REDCap software (Vanderbilt University, Tennessee, USA) (31, 32).

### Data analysis

Face validity tests were conducted after questionnaire completion by the respective study assessors (AS, ML) (31, 32). In case of unclear information, misspelling or missing data, study participants were contacted via email or telephone within 3 days filling out the questionnaire. Necessary corrections were documented directly in the respective questionnaire. A bottom-up, prevalence-based approach was used to retrospectively estimate the direct and indirect costs over the past 3 months for the study sample that underwent baseline testing until November 2025 (19, 33). All data from the iMCQ and iPCQ were presented in a non-aggregated form, along with the respective units consumed and the calculated unit prices. Costs were calculated as units consumed × unit price. Direct medical costs were monetised using the reference values as summarised in Supplementary file S1. This approach aligns with the health economic analysis plan of the BO2WL trial (20). Deviations from this health economic analysis plan concern the monetisation of a medical assistant’s appointments (e.g., for laboratory or blood pressure measurements), which are usually paid for through physicians´ consultations. To prevent double counting, these costs were excluded from the analysis. Medication costs were calculated per tablet consumed using the most recent speciality list for the reimbursement of medications in CH (34). Unpaid work was monetised using the “Proxy good method” by applying a hourly cost of CHF 20.34 (2025 CHF) for all unpaid work (35). In line with the health economic analysis plan and the recommendations of Swiss pharmacists, the medication price from three web-based pharmacies was used in case the medication was not listed. Nutritional supplements were also monetised using mean cost data from three web-based pharmacies. The human capital approach was used to calculate the indirect costs based on absenteeism and presenteeism data derived from the iPCQ questionnaire. The average annual income for CH in 2023 was CHF 94,507 per year with 8.4 h per day, 25 paid vacation days and 8 paid public holidays in Bern (CH) (36). This led to an hourly wage of CHF 49.56 before taxation, based on the following formula:


$$\text{Hourly wage}=\frac{\text{Annual income before}\;\mathrm{tax}}{\begin{array}{l} {}[52\times 5-(\begin{array}{l} \text{Paid vacation days}+\\ \text{Public holidays} \end{array})]\times \\ \text{Hours of standard workday} \end{array}}$$

Costs were adjusted to 2025 CHF and converted to 2025 Euros, using the following cost conversion formula and purchasing power parities (PPP) values as well as the GDP deflator index derived from the International Monetary Fund (37):


$$\text{Cost}2025\;\text{Euro}=\frac{\mathrm{GD}P_{2}\times \mathrm{PP}P_{2}}{\mathrm{GD}P_{1}\times \mathrm{PP}P_{1}}\times \text{Costoriginal}$$

The PPP_2_ and GDP_2_ values correspond to the target price year (2025) and currency (CHF or Euro), whereas PPP_1_ and GDP_1_ values represent the original price year (ranging from 2023 to 2025, depending on the date of baseline assessment) and the original currency (CHF). Discounting of costs was not necessary due to the retrospective data evaluation of 3 months duration (19). Total costs were calculated from a healthcare perspective, which included only direct medical costs, and from a societal perspective, considering direct medical, direct non-medical, and indirect costs. Distributional effects were assessed separately for individuals with overweight and obesity.

The uncertainty surrounding the cost-related data was addressed through non-parametric bootstrapping. This involved using the calculated cost from each main cost category (direct medical, direct non-medical, absenteeism, presenteeism, unpaid work) and conducting 5,000 iterations with the RStudio package “boot” (38, 39). All cost data were reported as mean values with their respective 95% confidence intervals (CI) values.

For the statistical data analysis RStudio version 4.5.0 (39) was used with the packages ‘tidyverse’ (version 2.0.0) for data manipulation (40), ‘eq5d’ (v.0.15.7) for utility calculation (41), boot (v.1.3–31) for bootstrapping (38, 39) and ggplot2 (v.3.5.2) for data visualisation (42). Patients or others affected by the study were not included in any step of the study development from planning to analysis.

## Results

Of the 53 participants assessed during baseline measurements of the BO2WL trial, 19 were male and 34 were female. Fifty-two participants were included in the calculation of the societal costs as no data were available for one participant for the health economic questionnaires. Most participants were classified as obese (*n* = 35), while 18 were overweight. On average, participants were 47.80 years old (SD: 10.10 years), with mean BMI values of 28.00 kg/m^2^ (SD: 1.19 kg/m^2^) for the overweight group and 32.90 kg/m^2^ (SD: 2.60 kg/m^2^) in the obesity group. The majority of the participants (41.50%, *n* = 22) held a degree from a school for higher vocational training, while 26.40% (*n* = 14) held a university degree and 18.90% (*n* = 10) a degree for intermediate vocational training.

Two of the participants who were overweight retired early without any physical or psychological reason and were therefore not working at the time of the baseline measurement. CLBP had persisted in patients on average 5.81 years (SD: 6.08 years) with variations in the overweight group (mean: 6.01 years, SD: 5.63 years) and the obesity group (mean: 5.72 years, SD: 6.38 years). The overall mean health utility index was 0.840 (SD: 0.133), with a reported mean subjective health of 73.10 (SD: 14.50) out of 100 on a numeric rating scale. Health utility scores for the overweight subgroup (mean: 0.876, SD 0.064) were similar to the obesity subgroup (mean: 0.822, SD: 0.153), which was also the case for subjective health status (overweight: 74.90, SD: 15.10; obesity: 72.10, SD: 14.30) (Table 2). Supplementary S2 includes the flowchart of participant recruitment.

**Table 2:** Study participants’ characteristics (all considered participants and stratified by BMI category).

	Overweight (*n* = 18)	Obesity (*n* = 35)	Overall (*n* = 53)
Sex			
Male	7 (38.9%)	12 (34.3%)	19 (35.8%)
Female	11 (61.1%)	23 (65.7%)	34 (64.2%)
Age			
Mean (SD)	47.6 (11.6)	47.8 (9.41)	47.8 (10.1)
BMI			
Mean (SD)	28.0 (1.19)	32.9 (2.60)	31.2 (3.22)
Marital status			
Single	4 (22.2%)	12 (34.3%)	16 (30.2%)
Married	13 (72.2%)	18 (51.4%)	31 (58.5%)
Dissolved civil partnership	0 (0%)	1 (2.9%)	1 (1.9%)
Divorced	1 (5.6%)	4 (11.4%)	5 (9.4%)
Work percentage			
Mean (SD)	69.4 (37.2)	79.8 (25.3)	76.3 (29.9)
Education			
Primary school or primary schools	0 (0%)	2 (5.7%)	2 (3.8%)
Recent vocational training	1 (5.6%)	0 (0%)	1 (1.9%)
General school of secondary level I	0 (0%)	1 (2.9%)	1 (1.9%)
Intermediate vocational training	4 (22.2%)	6 (17.1%)	10 (18.9%)
Higher general secondary school	2 (11.1%)	1 (2.9%)	3 (5.7%)
School for higher vocational training	6 (33.3%)	16 (45.7%)	22 (41.5%)
University	5 (27.8%)	9 (25.7%)	14 (26.4%)
Duration of weekly PA (min)			
Mean (SD)	173 (153)	109 (101)	131 (124)
Duration of LBP (months)			
Mean (SD)	72.1 (67.6)	68.6 (76.6)	69.8 (73.0)
Subjective health (NAS 0–100)			
Mean (SD)	74.9 (15.1)	72.1 (14.3)	73.1 (14.5)
Missing	1 (5.6%)	0 (0%)	1 (1.9%)
Utility (German value set)			
Mean (SD)	0.876 (0.0639)	0.822 (0.153)	0.840 (0.133)
Missing	1 (5.6%)	0 (0%)	1 (1.9%)

BMI, Body mass index; CLBP, Chronic low back pain; EQ-5D-5L, European Quality-of-Life-5 Dimensions-5 Levels; Max, maximum; min, minutes; Min, minimum; PA, Physical activity; SD, Standard deviation.

### Healthcare utilisation

Healthcare consultations within the three-month time horizon were reported by 34 of the 52 participants with 10 of them being overweight and 24 obese. Study participants consulted general practitioners (mean: 1.59 visits, SD: 0.96), physiotherapists (mean: 2.24 visits, SD: 3.24) and complementary medicine (mean: 1.18, SD: 3.18) more than once on average in the past 3 months. All other healthcare providers (e.g., occupational therapists, logopaedists, nutritionists, complementary medicine practitioners, psychologists, company doctors) were visited less than once or not at all during this period.

Moreover, participants did not receive therapy in an inpatient setting, including a day clinic, hospital stay, or daycare. Only one participant with overweight and two with obesity reported visits to an outpatient clinic (e.g., for a colonoscopy or mammography). While participants with overweight took on average 2.38 (SD: 1.51) different medications, individuals with obesity consumed 3.14 (SD: 1.88) different medications within the evaluated three-month period. Overall descriptives of the healthcare consumptions for all individuals are included in Supplementary file S3.

### Productivity loss

Presenteeism and absenteeism were reported by 67.31% (*n* = 35) and 30.77% (*n* = 16) of the individuals, respectively, while the other participant did not report any productivity loss. Presenteeism was higher among individuals with obesity (individuals with overweight: *n* = 12 vs. individuals with obesity: *n* = 23).

The mean number of absenteeism days over 3 months was lower in individuals with obesity (mean: 5.27 days, SD: 7.03) compared to those with overweight (mean: 12.80 days, SD: 19.20). However, both subgroups exhibited high variability (overweight range: 1.00 days to 47.00; obesity range: 1.00 days to 25.00).

Overall, the mean number of presenteeism days over 3 months was similar between the two subgroups, with an average of 11.40 days (SD: 15.90) among individuals with overweight and 11.80 days (SD: 11.50) for those with obesity. Nevertheless, some individuals reported extremely high presenteeism days (overweight range: 2.00 to 54.00 days; obesity range: 1.00 to 50.00 days). Workload fulfilment during presenteeism days was also similar between the groups, with individuals with overweight reporting 7.92 (SD: 1.83) and individuals with obesity 7.43 (SD: 2.02) on a scale from 0 to 10 (workload completely fulfilled). Again, some individuals displayed extreme values, with overall workload fulfilment among all participants ranging from 3.00 to 10.00.

Seventeen out of the 52 individuals (32.69%) reported impaired unpaid work, with an average of 18.40 (SD: 22.70) days during the three-month observation period. The individuals received on average 7.24 (SD: 11.00) hours of help during this time with individuals with obesity receiving more help (mean: 8.46 h, SD: 12.30 h) compared to individuals with overweight (mean: 3.25 h, SD: 3.86 h). Supplementary file S4 contains overall descriptives of productivity losses of all included individuals.

### Disaggregated cost data

On average, an individual with overweight or obesity and CLBP incurred direct medical costs of CHF 543.69 (95% CI = [382.80; 686.33]) and direct non-medical costs (e.g., transportation or nursing/domestic care) of CHF 19.39 (95% = [0.00; 38.63]) for the three-month time horizon. Indirect costs due to absenteeism, presenteeism and impaired unpaid work accounted for CHF 1,000.39 (95% CI = [2.63; 1,681.40]), CHF 549.47 (95% CI = [281.67; 774.67]) and CHF 745.56 (95% CI = [241.02; 1,436.80]), respectively within those 3 months. Overall, total societal costs amounted to CHF 2,850.44 (95% CI = [1,667.17; 3,829.17]), with healthcare costs contributing CHF 543.69 (95% CI = [382.80; 686.33]) over the three-month time horizon (Table 3).

**Table 3:** Disaggregated bootstrapped cost data, reported as mean (95% CI) (2025 CHF).

Cost category	Overweight	Obesity	Overall
Direct medical	474.61 [269.93; 667.10]	577.02 [355.51; 768.09]	543.69 [382.80; 686.33]
Direct non-medical	0.85 [0; 1.70]	28.4 [0; 56.76]	19.39 [0; 38.63]
Absenteeism	1,602.22 [0; 3,062.50]	705.91 [0; 1,206.28]	1,000.39 [2.63; 1,681.40]
Presenteeism	677.49 [0; 1,166.90]	486.74 [246.28; 701.70]	549.47 [281.67; 774.67]
Unpaid Work	436.71 [2.18; 1,237.28]	894.42 [232.81; 1,872.04]	745.56 [241.02; 1,436.80]
**Healthcare**	**474.61 [269.93; 667.10]**	**577.02 [355.51; 768.09]**	**543.69 [382.80; 686.33]**
**Societal**	**3,194.86 [399.25; 5,049.41]**	**2,691.96 [1,501.60; 3,646.68]**	**2,850.44 [1,667.17; 3,829.17]**

Healthcare costs: direct medical costs; Societal costs: all cost categories considered.

**Figure 1:**
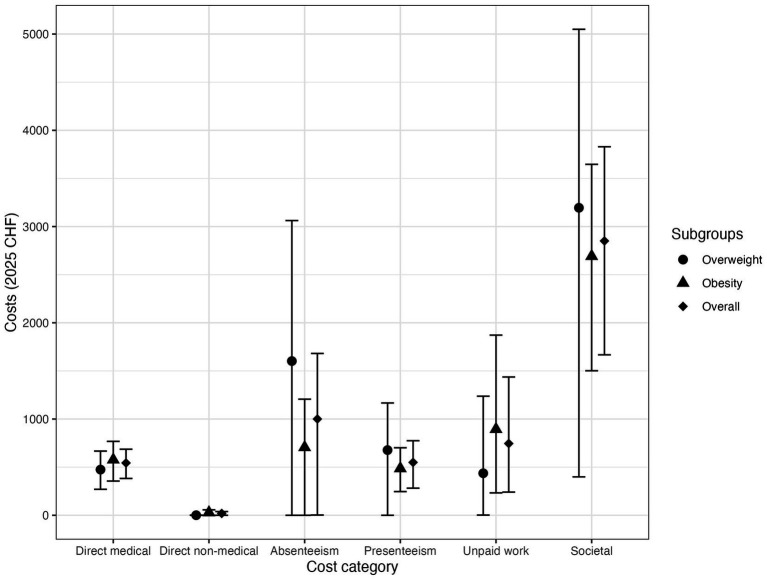
Boxplots of cost data (2025 CHF) in disaggregated form per BMI category. Mean values with 95% CI.

Average absenteeism and presenteeism costs were 154.31% or 54.41% higher in individuals with overweight, respectively, compared to individuals with obesity (Figure 1). Costs due to impaired unpaid work were on average 46.52% lower in individuals with overweight compared to those with obesity. The corresponding cost data, expressed in Euros, are provided in Supplementary file S5.

**Figure 2:**
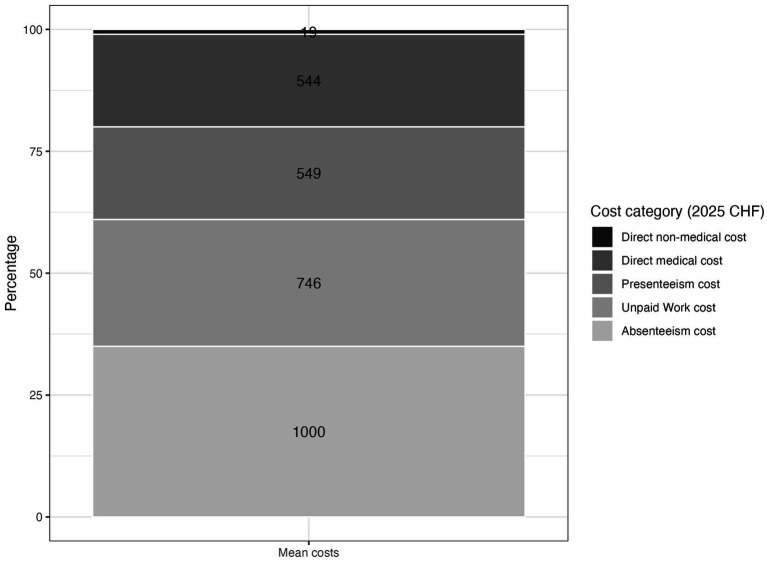
Stacked bar chart of percentages of mean societal cost data, values represent mean values per cost category (2025 CHF).

Most societal costs were attributed to lost productivity during both paid and unpaid work, primarily due to absenteeism (35.00%), impaired unpaid work (26.08%), and presenteeism (19.22%). In contrast, direct medical costs accounted for 19.02% of the total societal costs, while direct non-medical costs constituted 0.68% (Figure 2).

## Discussion

To the best of our knowledge, this cost of illness study is the first to evaluate the economic burden of individuals with overweight or obesity and CLBP in the Bern metropolitan area, CH. Over a three-month period, total societal costs amounted to CHF 2,850.44 per patient, with 80.30% attributed to indirect costs due to absenteeism, presenteeism and impaired unpaid work. Healthcare expenditures over the same period accounted for CHF 543.69 of the total societal costs.

Comparing economic results across different countries is challenging due to differences in healthcare systems and country-specific costs (43). In CH, no cost of illness study exists for individuals with overweight or obesity and CLBP, while existing cost of illness studies in the field of overweight or obesity in CH did not consider CLBP in their analysis (7, 15). One comparable study from Germany evaluated societal costs in patients with acute LBP (*n* = 643) and CLBP (*n* = 451) over 6 months using a bottom-up approach (8), without BMI restrictions. Societal costs were lower than in the present study with €1,789.81 (95% CI = [1,470.0; 2,202.0]; CHF 4,052.50, 95% CI = [3,328.39; 4,985.79]) per patient over the 6 months (8).

Indirect costs were the main cost driver in the present study, accounting for 80.30% of the total societal costs (absenteeism costs: 35.00%, presenteeism costs: 19.22%, Unpaid work costs: 26.08%) which aligns with findings from other countries in this field. In the aforementioned German study, absenteeism costs accounted for 52%, while the costs of presenteeism and unpaid work were not evaluated (8). Literature on individuals with acute LBP from CH (5), the United States (6) and Sweden (10, 44) reported productivity losses accounting for 42 to 78% of the total societal costs.

A register-based cross-sectional study in CH examined factors associated with productivity loss in 161 individuals with chronic MSK pain prior to participation in a chronic care management program (45). Productivity loss was measured prospectively using the iPCQ. Statistically significant associations (all *p* < 0.01) were found between productivity loss and stress in professional situations (*r* = 0.59), difficulties in balancing work and family responsibilities

(*r* = 0.19), residence status (*r* = −0.20), and sex (*r* = 0.21) with men reporting higher productivity losses (45). Interestingly, no statistically significant association was found with average pain intensity in the week prior to the evaluation (*r* = −0.06) in these individuals with chronic MSK pain (45). This finding contrasts with the results of a cross-sectional study of 2086 individuals with CLBP in five European countries (Germany, France, the United Kingdom, Italy, Spain), which reported statistically significantly higher absenteeism among those individuals with moderate to high pain levels compared to those experiencing low pain levels (8.81% vs. 4.75%, *p* < 0.01) (13).

Cost comparisons between different cost of illness papers are challenging due to the variety of methodological approaches, such as the human capital approach or the friction cost method (46). These methods lead to differences in the calculated indirect costs (46). For example, a Swiss study from 2005 evaluated the costs of individuals with acute LBP from a societal perspective, with variations in the indirect costs from €1,571 to €2,926 (CHF: 2,790.62 to 5,197.55) depending on the use of the human capital approach or friction cost method, respectively (5). These accounted for 46.0 to 61.4% of total costs (5). Similar variations in cost calculations were evident in the aforementioned German study of individuals with acute LBP and CLBP (8).

Difference in daily wages also affects the calculation of the productivity costs as the German study used a daily wage of CHF 245.39 (€108.38) (8), while the present cost of illness study used a daily wage of CHF 416.30. Moreover, productivity is often assessed using questionnaires such as the iPCQ (23, 24, 47). For example, questions related to presenteeism include “How many working days were you affected by physical complaints or psychological problems?” (23, 24). Such questions may result in subjective reporting, potentially influenced by recall bias, since respondents are required to consider the past 3 months, or by social desirability bias (47). The collection of these kind of data involves a trade-off between data quality and participant burden (47). For example, one study recommended that retrospective assessments should not exceed 2 months to reduce the risk of recall bias (48). This may also have influenced the data on healthcare costs for the individuals included in the present study, which totalled CHF 543.69, comparable to that of the general Swiss population (1). In 2022, CHF 50 was spent on cost-sharing with health insurance, while out-of-pocket costs amounted to CHF 138 per month, leading to a total of CHF 564 when extrapolated over 3 months (1). However, the reported healthcare costs for the general CH population encompasses all age groups, with 52% of the healthcare costs attributed to individuals over 61 years of age (1). In contrast, the present cost of illness study included individuals only up to the age of 65 years.

Absenteeism data for all included individuals in this study were 44% higher compared to the Swiss average absenteeism, with 2.35 days (SD: 7.42, Supplementary file S4) compared to 1.63 days per 3 months (49). This result is not surprising as higher BMI values are related to lower productivity (50–53). Absenteeism (126%) was higher in individuals with overweight compared to those with obesity. The small sample size of individuals with overweight (*n* = 17) and the right-skewed distribution of the cost-related data (54) may have contributed to this pattern. For instance, removing one individual with overweight and 47 days of absenteeism would lower the absenteeism costs for the individuals with overweight from 1,000.39 [2.63; 1,681.40] to CHF 454.66 (95% CI = [35.49; 922.64]).

Several limitations of the present study should be noted. Firstly, the sample size of 53 individuals was small, which reduces the precision of the study results. This limited sample size partly reflects the difficulty of accessing individuals with both overweight or obesity and CLBP. By applying bootstrapping, the most appropriate method available was used to maximise the robustness of the study results given these constraints. The exclusion of individuals with uncontrolled comorbidities in the present cost of illness study may limit the applicability of the results to the broader population of individuals with overweight or obesity and CLBP. Such comorbidities may be associated with higher healthcare utilisation, increased medication usage and greater absenteeism or presenteeism losses, resulting in higher societal costs than those estimated in this study. However, excluding cases with severe health conditions is common in chronic pain research as evaluated in a systematic review (55). For example, 59 % of the randomised controlled trials in individuals with chronic pain excluded individuals with severe depressive symptoms although depressive symptoms were evaluated in the trial (55).

Secondly, individuals with surgical or pharmacological treatment for overweight or obesity to induce weight loss were excluded. Including them might have increased especially the direct medical costs (e.g., medication costs) or influenced productivity loss positively (e.g., reduction of type II diabetes symptoms). The same applies to individuals starting new pharmacological treatments for any other health condition within the last 6 months or with unstable health conditions, e.g., unstable arterial hypertension.

Thirdly, costs could not be directly linked to CLBP or to being overweight or obese, as healthcare consultations could not be linked to either condition. Comorbidities are related to higher costs in individuals with LBP (56). For example, the costs for individuals with LBP and comorbid diabetes or depression were USD 2,937 (CHF: 4,408) or USD 1,430 (CHF: 2,146) higher compared to those without these comorbidities (56). Consequently, the cost estimates in the present study may reflect the combined economic burden of individuals with overweight or obesity, CLBP, and any other existing comorbidities, rather than costs specific to CLBP alone. Distinguishing costs based on the health conditions is difficult, especially when one therapy has beneficial effects on more than one condition, which is the case for exercise therapy. It has positive impacts on, e.g., CLBP (57), arterial hypertension (58, 59) and overweight or obesity (60).

Fourthly, this analysis did not include intangible costs, such as costs related to stigma, which is often experienced by individuals with overweight or obesity and/or chronic pain (61–63). Based on an evaluation of individuals with overweight or obesity in Germany, the intangible costs were estimated at €13,853 (CHF 22,375) and €42,450 (CHF 68,565) per person per year (64). Each increase in BMI leads to an additional intangible cost of €2,553 (CHF 4,124) per year (64). However, this cost category is extremely difficult to quantify and presents a risk of double counting (25–27), as the positive or negative effects of an intervention might reduce or increase not only pain but also associated intangible costs. This is why these costs were not evaluated, notwithstanding their societal impact.

To our knowledge, this is the first study to assess the societal costs of patients with overweight or obesity and CLBP, emphasising the novel nature of the present study. Key strengths of this study were the use of non-parametric bootstrapping to account for the self-reported and right skewed data and thus reducing reporting or recall bias. Additionally, the data were reported comprehensively by also monetising aspects, such as supplement usage, the adherence to the CHEERS guideline and the trial-based nature of this cost of illness evaluation.

In conclusion, individuals with overweight or obesity and CLBP in the metropolitan area of Bern face a substantial financial burden, with most of the societal costs related to productivity losses. Targeted interventions should improve the health status of those affected and reduce their economic burden. Future randomised controlled trials with health economic evaluations from a societal perspective should consider health insurance data and employ standardised methodology to enhance the comparability of the findings. Furthermore, the results highlight the potential value of more coordinated policy responses, such as workplace health initiatives, to reduce productivity losses and lower the high indirect costs.

## Data Availability

The raw data supporting the conclusions of this article will be made available by the authors, upon reasonable request.
