# Supplementary material for: Cost of illness analysis in individuals with overweight or obesity and chronic low back pain in the Bern metropolitan area (the BO2WL trial)
Source: Front Public Health. 2026 Jan 13;13:1705891. doi: 10.3389/fpubh.2025.1705891 (PMC12835357; doi:10.3389/fpubh.2025.1705891)
Supplement: Supplementary file 1 [file Data_Sheet_1.pdf]

S1: References for valuation of cost-related data (costs expressed as 2025 Swiss Francs).

Appointments/ Care/ Transportation	Price (CHF)	Reference
General practitioner	155.00	Federal Office of Public Health
Medical assistant	0.00	Valued through medical doctor consultations
Social worker	41.05	
Physical therapist (general appointment)	49.44	General tariffs
Physical therapist (1st appointment)	74.16	General tariffs
Occupational therapist	52.80	General tariffs
Logopaedist (general appointment)	82.68	General tariffs
Logopaedist (added costs for all appointments)	82.68	General tariffs
Nutritionist (1st appointment)	107.91	General tariffs
Nutritionist (2-6th appointment)	83.93	General tariffs
Nutritionist (≥7th appointment)	69.76	General tariffs
Complementary medicine	148.67	Average price from three complementary medicine professionals (156 CHF, 150 CHF, 140 CHF)
Psychological care	178.02	Swiss remuneration system for medical staff
Outpatient clinic appointments		Reported costs by participants and health insurance claims
Proctology	261.25	
Colonoscopy	764.50	
Mammography	166.20	
Gynaecologist	278.35	
Neurology	155.00	
Company doctor	135.00	Reported prices by three company doctors
Domestic care	21.93	General tariffs
Personal care	64.17	Nursing service regulations Article 7a
Nursing care	64.17	Nursing service regulations Article 7a
Family care	37.90	Pflegewegweiser
Transportation (costs per km)		
Car	0.76	Average costs per km
Pedestrian	0.00	Not monetised
Public transport	0.43	Average costs per km for train travel from Bern to Zurich: 53CHF, 122km

S2: Flowchart of study participants

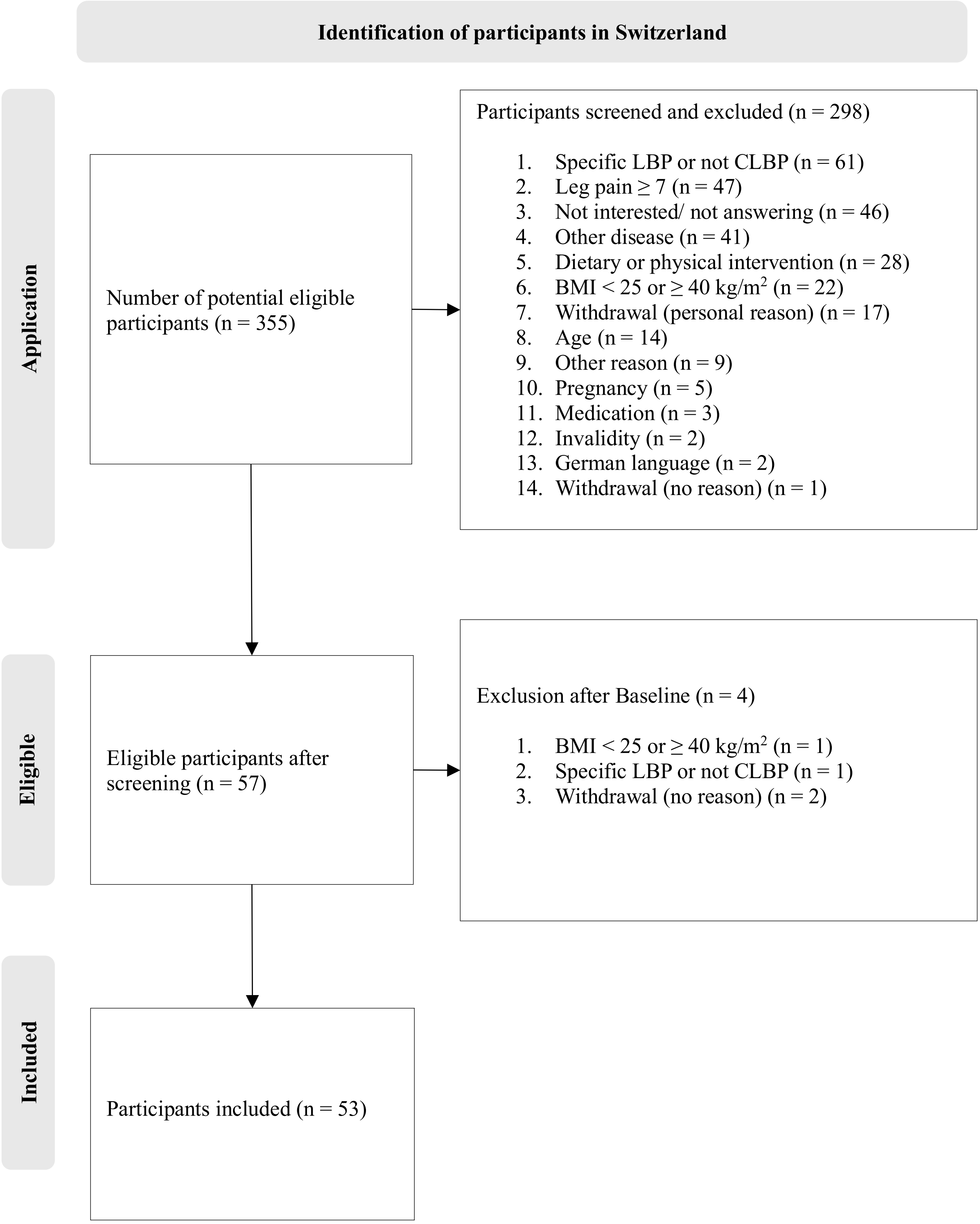

Abbreviations: BMI: Body Mass Index, CLBP: chronic low back pain, CNLBP: chronic non-specific low back pain, LBP: low back pain

S3: Descriptive data iMCQ.

	**Overweight** (N=17)	**Obesity** (N=35)	**Overall** (N=52)
**GP**			
Mean (SD)	0.941 (1.14)	1.09 (1.07)	1.04 (1.08)
**Medical assistant**			
Mean (SD)	0.824 (2.92)	0.886 (1.57)	0.865 (2.08)
**Social worker**			
Mean (SD)	0 (0)	0.0571 (0.338)	0.0385 (0.277)
**Physical therapist**			
Mean (SD)	2.06 (3.82)	1.17 (2.19)	1.46 (2.82)
**Occupational therapist**			
Mean (SD)	0 (0)	0.257 (1.12)	0.173 (0.923)
**Logopaedist**			
Mean (SD)	0 (0)	0 (0)	0 (0)
**Nutritionist**			
Mean (SD)	0 (0)	0.114 (0.404)	0.0769 (0.334)
**Complementary medicine**			
Mean (SD)	0.118 (0.485)	1.09 (3.14)	0.769 (2.62)
**Psychological care**			
Mean (SD)	0.118 (0.485)	0.0571 (0.338)	0.0769 (0.388)
**Company doctor**			
Mean (SD)	0.0588 (0.243)	0.0571 (0.338)	0.0577 (0.308)
**Care**			
No	16 (94.1%)	35 (100%)	51 (98.1%)
Yes	1 (5.9%)	0 (0%)	1 (1.9%)
**Medication**			
No	4 (23.5%)	6 (17.1%)	10 (19.2%)
Yes	13 (76.5%)	29 (82.9%)	42 (80.8%)
**Number of medications**			
Mean (SD)	2.38 (1.61)	2.90 (1.78)	2.74 (1.73)
**Hospital**			
No	16 (94.1%)	33 (94.3%)	49 (94.2%)
Yes	1 (5.9%)	2 (5.7%)	3 (5.8%)
**Emergency department**			
Mean (SD)	0 (0)	0 (0)	0 (0)
**Ambulance**			
Mean (SD)	0 (0)	0 (0)	0 (0)
**Outpatient clinic**			
No	16 (94.1%)	33 (94.3%)	49 (94.2%)
Yes	1 (5.9%)	2 (5.7%)	3 (5.8%)
**Day clinic**			
No	17 (100%)	35 (100%)	52 (100%)
Yes	0 (0%)	0 (0%)	0 (0%)
**Day care**			
No	17 (100%)	35 (100%)	52 (100%)
Yes	0 (0%)	0 (0%)	0 (0%)
**Hospital stays**			
No	17 (100%)	35 (100%)	52 (100%)
Yes	0 (0%)	0 (0%)	0 (0%)
**Other inpatient stays**			
No	17 (100%)	35 (100%)	52 (100%)
Yes	0 (0%)	0 (0%)	0 (0%)

S4: Descriptive data iPCQ.

	Overweight (N=17)	Obesity (N=35)	Overall (N=52)
**Working (hours/week)**			
Mean (SD)	31.6 (13.4)	31.6 (13.7)	31.6 (13.5)
**Workdays (week)**			
Mean (SD)	3.65 (1.61)	3.99 (1.55)	3.88 (1.56)
**Absenteeism**			
No	10 (58.8%)	21 (60.0%)	31 (59.6%)
Yes	5 (29.4%)	11 (31.4%)	16 (30.8%)
No paid work	2 (11.8%)	3 (8.6%)	5 (9.6%)
**Absenteeism (days)**			
Mean (SD)	3.76 (11.3)	1.66 (4.55)	2.35 (7.42)
**Presenteeism**			
No	3 (17.6%)	9 (25.7%)	12 (23.1%)
Yes	12 (70.6%)	23 (65.7%)	35 (67.3%)
No paid work	2 (11.8%)	3 (8.6%)	5 (9.6%)
**Presenteeism (days)**			
Mean (SD)	8.06 (14.2)	7.77 (10.9)	7.87 (11.9)
**Presenteeism workload fulfilment**			
Mean (SD)	7.92 (1.83)	7.43 (2.02)	7.60 (1.94)
Missing	5 (29.4%)	12 (34.3%)	17 (32.7%)
**Impaired unpaid work**			
No	13 (76.5%)	22 (62.9%)	35 (67.3%)
Yes	4 (23.5%)	13 (37.1%)	17 (32.7%)
**Impaired unpaid work (days)**			
Mean (SD)	2.88 (7.30)	7.51 (18.0)	6.00 (15.4)
**Help during unpaid work (hours)**			
Mean (SD)	0.765 (2.19)	3.14 (8.39)	2.37 (7.05)

S5: Disaggregated bootstrapped cost data (2025 EUR): Mean values with 95% CI values.

Cost category	Overweight	Obesity	Overall
Direct medical	348.31 [198.10; 489.58]	423.47 [260.90; 563.69]	399.01 [280.93; 503.69]
Direct non-medical	0.62 [0; 1.25]	20.84 [0; 41.66]	14.23 [0; 28.35]
Absenteeism	1,175.85 [0; 2,247.53]	518.06 [0; 885.27]	734.17 [1.93; 1,233.96]
Presenteeism	497.20 [0; 856.37]	357.21 [180.74; 514.97]	403.25 [206.71; 568.52]
Unpaid Work	320.50 [1.6; 908.02]	656.4 [170.86; 1,373.87]	547.16 [176.88; 1,054.45]
**Healthcare**	**474.61 [269.93; 667.10]**	**577.02 [355.51; 768.09]**	**543.69 [382.80; 686.33]**
**Societal**	**2,344.67 [293.00; 3,705.70]**	**1,975.60 [1,102.01; 2,676.25]**	**2,091.90 [1,223.52; 2,810.18]**
